# Mechanism of Green Tea Peptides in Lowering Blood Pressure and Alleviating Renal Injury Induced by Hypertension Through the Ang II/TGF-β1/SMAD Signaling Pathway

**DOI:** 10.3390/nu17081300

**Published:** 2025-04-08

**Authors:** Lulu Li, Shili Sun, Xingfei Lai, Qiuhua Li, Ruohong Chen, Zhenbiao Zhang, Mengjiao Hao, Suwan Zhang, Lingli Sun, Dongli Li

**Affiliations:** 1 School of Biotechnology and Health Sciences, Wuyi University, Jiangmen 529020, China; lll9423@126.com; 2 Tea Research Institute, Guangdong Academy of Agricultural Sciences/Guangdong Key Laboratory of Tea Resources Innovation & Utilization, Guangzhou 510640, China; sunshili@zju.edu.cn (S.S.); laixingfei@gdaas.cn (X.L.); liqiuhua@gdaas.cn (Q.L.); chenruohong@gdaas.cn (R.C.); zhangzhenbiao@gdaas.cn (Z.Z.); haomengjiao@gdaas.cn (M.H.); swzhang1502@163.com (S.Z.); 3 International Healthcare Innovation Institute (Jiangmen), Jiangmen 529040, China

**Keywords:** tea polypeptide, hypertension, renal injury, TGF-β/Smad signaling

## Abstract

**Background/Objectives:** The kidney plays a crucial role in regulating normal blood pressure and is one of the major organs affected by hypertension. The present study aimed to investigate the hypotensive and renoprotective effects of four specific green tea peptides extracted from green tea dregs on spontaneously hypertensive rats (SHRs) and to investigate the underlying mechanisms. **Methods:** Four specific green tea peptides (40 mg/kg) were gavaged to SHRs for 4 weeks, and blood pressure, renal function, renal pathological changes, renal tissue fibrosis indexes, and inflammation indexes were examined in SHRs to analyze the role of the four green tea peptides in alleviating hypertension and its renal injury. **Results:** The results showed that the four TPs significantly reduced systolic and diastolic blood pressure (20–24% and 18–28%) in SHR compared to the model group. Meanwhile, gene levels and protein expression of renal fibrosis-related targets such as phospho-Smad2/3 (*p*-Smad2/3) (26–47%), Sma- and Mad-related proteins 2/3 (Smad2/3) (19–38%), transforming growth factor-β1 (TGF-β1) (36–63%), and alpha-smooth muscle actin (alpha-SMA) (58–86%) were also significantly reduced. In addition, the reduced expression levels of medullary differentiation factor 88 (MyD88) (14–36%), inducible nitric oxide synthase (iNOS) (58–73%), and nuclear factor-κB p65 (NF-kB p65) (35–78%) in kidneys also confirmed that TPs attenuated renal inflammation in SHR. Therefore, green tea peptides could attenuate the fibrosis and inflammatory responses occurring in hypertensive kidneys by inhibiting the Ang II/TGF-β1/SMAD signaling pathway and MyD88/NF-κB p65/iNOS signaling pathway. **Conclusions:** The results showed that green tea peptides may be effective candidates for lowering blood pressure and attenuating kidney injury.

## 1. Introduction

Hypertension is widely recognized as an important non-communicable health condition. Recent estimates indicate that hypertension impacts nearly one-third of the global population (31.1%, 1.39 billion individuals) [1]. Among hypertensive patients, 16% are reported to have chronic kidney disease [2]. Renal injury induced by hypertension is recognized as one of the consequences of poorly controlled long-term hypertension and is a significant contributing factor to end-stage renal disease (ESRD) [3]. There is an increasing body of evidence indicating that prolonged hypertension adversely affects renal tubular cells, resulting in tubulointerstitial fibrosis, a key pathological process in renal injury induced by hypertension [4]. Research indicates that elevated pulse pressure stiffens small arteries and activates the renin–angiotensin–aldosterone system (RAAS), which is crucial in the regulation of blood pressure [5]. The activation of RAAS is crucial in hypertension-induced kidney injury [6,7]. Among its components, angiotensin II (Ang II) is a significant driver of hypertension-induced fibrosis [8,9,10]. Over time, Ang II causes tubular damage, glomerulosclerosis, and renal fibrosis via a TGF-β1-dependent signaling pathway, ultimately impairing renal autoregulation [11,12,13]. In addition, RAAS cooperates with TGF-β1 to facilitate epithelial-to-mesenchymal transition (EMT) in renal epithelial cells and has a pro-fibrotic effect on renal tissues [14]. Current treatments for hypertensive nephropathy encompass the use of calcium channel blockers, angiotensin-converting enzyme (ACE) inhibitors, and angiotensin receptor blockers [15]. However, long-term use of these medications is prone to cause significant side effects such as coughing, dizziness, and confusion [16]. In recent years, food-borne ACE inhibitors from natural sources have gained attention due to their safety, high efficacy, and excellent bioavailability [17,18,19]. As a result, it is highly significant to develop food-based ACE inhibitors that can help reduce kidney damage caused by hypertension while minimizing side effects.

Tea initially emerged in China for its medicinal value. The bioactive constituents of tea have exhibited a range of beneficial effects in the prevention of metabolic disorders, such as diabetes and hepatic metabolic dysfunction [20], neurodegenerative diseases [21], cardiovascular conditions [22], and inflammatory diseases [23]. Green tea is the most widely produced tea in China, accounting for about 65% of the country’s total tea production. It serves as a highly valuable source of plant-based proteins, as it contains a wide range of amino acids and is rich in non-water-soluble proteins, which account for 16–24% of the dry matter weight of tea leaves [24]. Studies have shown that green tea and selenium-enriched green tea are able to significantly improve vascular function by regulating intestinal flora, lowering levels of blood pressure, oxidative stress, inflammation, and endothelial dysfunction, as well as protecting cardiac, hepatic, and renal tissues [25]. Tea dregs protein was extracted by the alkaline method and combined with enzymatic hydrolysis. The obtained hydrolysate of green tea dregs protein showed significant ACE inhibitory activity and showed effective antihypertensive activity in vivo [26]. Furthermore, by targeting the TGF-β1 signaling pathway, green tea peptides may decrease blood glucose levels and treat diabetic nephropathy [27]. Specifically, TGF-β1 is upregulated in the glomerulus and stroma of the fibrotic kidney, which further aggravates fibrosis [28]. Research targeting TGF-β1 and its associated Smad2/3 signaling pathway has demonstrated a reduction in the phosphorylation of Smad2/3, effectively inhibiting renal fibrosis [29]. Consequently, we propose that the TGF-β1/Smad2/3 signaling pathway may be integral to the pathophysiology of advanced renal conditions, including hypertensive glomerulosclerosis and tubular fibrosis.

The proteins of green tea digest isolated from green tea dregs were previously extracted and analyzed for the amino acid sequence and molecular weight of tea ACE inhibitory peptide by LC-MS/MS and obtained four ACE inhibitory peptides, three 7-peptide structures, LAEQAER, VECTIPK, and MASLALK, and one 11-peptide structure, DAYVGDEAQSK, were obtained from green tea residues. The study showed that the hypotensive activity of the peptides was related to the sequence of the peptides and the amino acid species. Moreover, food-derived ACE inhibitory peptides are mostly oligopeptides containing 2–12 amino acid residues and have a high content of hydrophobic amino acids (hydrophobic amino acids leucine, valine, alanine, tryptophan, tyrosine, proline, and phenylalanine) [30,31]. The four peptides in this study have a high content of hydrophobic amino acids, among which MASLALK has the highest hydrophobic amino acid content, with the proportion of hydrophobic amino acids reaching 71.4%. Therefore, we chose these four ACE inhibitory peptides to explore their antihypertensive effects and effects exerted in vivo.

We used a spontaneous hypertensive rat (SHR) model to systematically investigate for the first time that four food-derived ACE inhibitory peptides obtained from green tea dregs not only alleviate hypertension symptoms but also ameliorate renal fibrosis and inflammatory responses. Green tea peptides have the unique advantage of being natural and safe compared to existing drug-based ACE inhibitors. Its food-borne characteristics make it easier to take for long periods of time with fewer side effects, providing a new strategy for the prevention and treatment of hypertension and its complications, as well as new insights into the innovative application of tea waste resources.

## 2. Materials and Methods

### 2.1. Green Tea Peptide Preparation

The green tea was produced from the Jin Xuan cultivar, which was cultivated at the Tea Research Institute of the Guangdong Academy of Agricultural Sciences. The fresh tea leaves underwent an initial heating process at a temperature of 230 °C to inactivate endogenous enzymes. Subsequently, the leaves were subjected to a kneading process lasting 30 min, followed by direct drying to produce green tea. The specific processing methods for green tea peptides are detailed in the literature [26]. Green tea protein hydrolysate was obtained by enzymatic hydrolysis, alkali extraction, acid precipitation, desalination, decolorization, and acid protease enzymatic hydrolysis of green tea residue. The green tea protein hydrolysate was further purified by ultrafiltration, gel chromatography, and other separation techniques to obtain small molecular peptides. Finally, four peptides were identified by matrix-assisted laser desorption ionization time-of-flight mass spectrometry (MALDI-TOF/MS) (Bruker, Daltonics, Germany) and two-dimensional mass spectrometry (2D-MS) (Thermo Fisher, Scientific, Shanghai, China). They were characterized using Eksigent Nano LC-MS/MS (Thermo Fisher, Scientific, Shanghai, China). The amino acid sequence of TP1 is LAEQAER (Leu-Ala-Glu-Gln-Ala-Glu-Arg), molecular weight 815.89, purity 98.3%. The sequence of TP2 is VECTIPK (Val-Glu-Cys-Thr-Ile-Pro-Lys), molecular weight 788.96, purity 98.6%. TP3 sequence is DAVGDEAQSK (Asp-Ala-Tyr-Val-Gly-Asp-Glu-Ala-Gln-Ser-Lys), molecular weight of 1182.22, purity of 98.0%. The sequence of TP4 is MASLALK (Met-Ala-Ser-Leu-Ala-Leu-Lys), molecular weight 732.95, purity 99.0% [27]. These four peptides have higher activity under acidic conditions, with good resistance to digestion and stability.

### 2.2. Animals and Treatment

All rats were housed and cared for according to the Animal Care and Use Guidelines approved by the Animal Care and Use Committee of the Tea Research Institute of Guangdong Academy of Agricultural Sciences (No. 2023002). Animal handling followed the Declaration of Helsinki [32]. Guangzhou Chasi Rui Hua Biotechnology Co provided 42 male spontaneously hypertensive rat (SHRs) and 7 male Wistar Kyoto rats (WKYs), all at 7–8 weeks of age. All animals were housed in a pathogen-free laboratory maintained at a temperature of 23.2 °C, a relative humidity of 55%, and a 12 h light/dark cycle, with ad libitum access to food and water. Following a one-week acclimation period, standard WKY were used as the control group (*n* = 7). SHRs were randomly assigned to the following groups (*n* = 7 per group): (1) model group, receiving distilled water; (2) positive control group, treated with captopril dissolved in distilled water at a dosage of 10 mg/kg body weight; (3) peptide-1 group, receiving TP1 dissolved in distilled water at 40 mg/kg body weight; (4) peptide-2 group, receiving TP2 dissolved in distilled water at 40 mg/kg body weight; (5) peptide-3 group, receiving TP3 dissolved in distilled water at 40 mg/kg body weight; and (6) peptide-4 group, receiving TP4 dissolved in distilled water at 40 mg/kg body weight. All treatments were administered via oral gavage once daily for 4 weeks. The normal control group received an equal volume of distilled water. The rats were weighed daily, water and food consumption were assessed bi-daily, and blood pressure readings were recorded weekly.

### 2.3. Blood Pressure Measurements

The blood pressure of the rats was measured by a non-invasive tail blood pressure device (Yaokun, Hefei, China) once a week. The rats were placed in a specially designed fixator, and a pressure cuff was inserted through the tail of the rat and fixed near the base of the tail. The high-sensitivity pulse sensor is placed at the upper 1/3 of the tail, and the surface of the sensor is closely attached to the ventral side of the tail for fixation. Increase the pressure with a rubber ball until the pressure in the pressure cuff is raised enough to eliminate the pulse signal. At this point, continue to pressurize about 20 mmHg, and then gradually release until the pulse signal returns to its original level. Systolic blood pressures (SBPs) and diastolic blood pressures (DBPs), as well as heart rate (HR), were measured 3–5 times consecutively, and the average of these readings was recorded as the final value.

### 2.4. Renal Function and Histopathology

Upon the conclusion of the study period, each rat was anesthetized intraperitoneally with sodium pentobarbital at a dose of 30 mg/kg, and blood samples were collected from the abdominal aorta to obtain serum. Serum levels of creatinine (CRE, C011-2-1, Nanjing Jiancheng, Nanjing, China) and urea nitrogen (BUN, C013-2-1, Nanjing Jiancheng, Nanjing, China) were measured using the kit. Additionally, the levels of rat ACE (MM-0212R1, detection range 5 g/mL–180 ng/mL, assay difference 7.3%, intra-assay difference 5.6%), angiotensin I (Ang I, MM-0394R1, detection range 4–140 pg/mL, assay range 7.5%, intra-assay difference 5.3%), and Ang II (MM-0211R1, detection range 15–400 ng/mL, 7.3% between batches and 5.4% within batches) were measured using ELISA kits (Meimian, Wuhan, China) to determine their levels in the kidneys. Kidney tissue was fixed in 4% paraformaldehyde (PFA) for more than 24 h and then embedded in paraffin. All sections (5 μm) were stained using hematoxylin and eosin (H&E, Beyotime, Shanghai, China) as well as Masson trichrome staining. The H&E injury score was determined by the proportion of renal tubules exhibiting damage, which may include tubular dilation or atrophy, tubular necrosis, loss of brush border, or the presence of casts. The degree of damage was graded using the following scale: 0, normal; 1, <10%; 2, 11–25%; 3, 26–75%; and 4, >75% of the observed tubules [33]. Masson trichrome staining was performed using a trichrome staining kit (Masson, Solarbio, Beijing, China) and examined under an inverted microscope. Collagen fibers, cytoplasm, and nuclei were stained blue, pink, and dark brown, respectively.

### 2.5. Real-Time Quantitative Polymerase Chain (RT-qPCR) Analysis

Rat kidney RNA was extracted using the E.Z.N.A^®^ Total RNA Kit I (Omega, Guangzhou, China) and RNA concentration was determined in a microvolume spectrophotometer (KRIRO, Beijing, China). RNA was reverse transcribed to cDNA using the RNA Reverse Transcription Kit (TOYOBO, Osaka, Japan) and RT-qPCR was performed on a real-time PCR instrument (Roche, Suzhou, China LightCycler 96) using Hieff^®^ qPCR SYBR Green Master Mix (No Rox) (YEASEN, Shanghai, China). A three-step amplification procedure was used: pre-denaturation for 5 min at 95 °C, 10 s at 40 cycles at 95 °C, 20 s at 60 °C, and 20 s at 72 °C. The data of 2^−ΔΔCt^ were used for calculation and analysis. Gene expression was normalized to GAPDH as the internal control. The primer sequences corresponding to the target genes are presented in Table 1.

### 2.6. Western Blotting

Weigh 40 mg of kidney tissue sample into a homogenate tube and homogenize with 400 μL of lysate (980 μL of radioimmunoprecipitation assay (RIPA, Beyotime, Shanghai, China), 10 μL of phenylmethanesulfonyl fluoride (PMSF), and 10 μL of Phosphatase Inhibitor Cocktail (Beyotime, Shanghai, China). Centrifuge the homogenate at 4 °C for 20 min at 18,506× *g* and collect the supernatant. The protein content in the supernatant was determined using Bidicinchoninic acid (BCA, Thermo, Waltham, MA, USA). An equal amount of each sample was mixed with a quarter volume of loading buffer 4× and denatured in a metal bath at 98 °C for 5 min. Proteins were separated using polyolefin-neutral gel electrophoresis (80–120 V) and then transferred to polyvinylidene fluoride (PVDF) membranes. At room temperature, seal for 2 h with 5% skim milk. After 2 h of shaking with 5% skimmed milk at room temperature, the primary antibody was prepared with a primary antibody dilution containing 5% bovine serum albumin (BSA). The specific antibody dilution ratios are listed as follows: Glyceraldehyde-3-phosphate dehydrogenase, Glyceraldehyde-3-phosphate dehydrogenase (GAPDH, CST, 5174S, Danvers, MA, USA, Rabbit, 1:1000), angiotensin-converting enzyme (ACE, Boster, PB9124, Wuhan, China, Rabbit, 1:1000), angiotensin II (Ang II, Bioss, bs-0587R, Beijing, China, Rabbit, 1:1000), transforming growth factor-β1 (TGF-β1, Boster, BA0290, Wuhan, China, Rabbit, 1:1000), Sma- and Mad-related proteins 2/3 (Smad2/3, CST, 8685S, Danvers, MA, USA, Rabbit, 1:1000), phosphorylated-Smad2/3 (*p*-Smad2/3, CST, 8828S, Danvers, MA, USA Rabbit, 1:1000), α-smooth muscle actin (α-SMA, Boster, BM0002, Wuhan, China, Mouse, 1:1000), medullary differentiation factor 88 (MyD88, CST, 4283S, Danvers, MA, USA, Rabbit, 1:1000), inducible nitric oxide synthase (iNOS, Abcam, ab15323, Cambridge, UK, Rabbit, 1:1000), and nuclear factor-κB p65 (NF-κB p65, CST, 8242S, Danvers, MA, USA, Rabbit 1:1000). The membrane was incubated overnight with the above-mentioned antibody at 4 °C and then incubated for 50 min by oscillating at 37 °C with the secondary antibody. After washing the film with TBST, add an electrochemical luminescence (ECL) reagent for 2–3 min. The films were exposed from 10 s to 5 min (exposure time was adjusted based on light intensity), and the bands were scanned and quantified for intensity using ImageJ software (Windows 64-bit). Results were normalized to the density of GAPDH bands.

### 2.7. Statistical Analysis

Statistical analyses were performed using GraphPad Prism 8.0 (San Diego, CA, USA) software. The Kruskal–Wallis test was used to validate between group differences, and the Mann–Whitney U test was used to complete post hoc comparisons. Results are presented as SD ± mean and *p* < 0.05 indicates statistical significance. Means with different letters indicate significant differences between groups.

## 3. Results

### 3.1. Green Tea Peptide Attenuated Hypertension in SHRs

**Figure 1:**
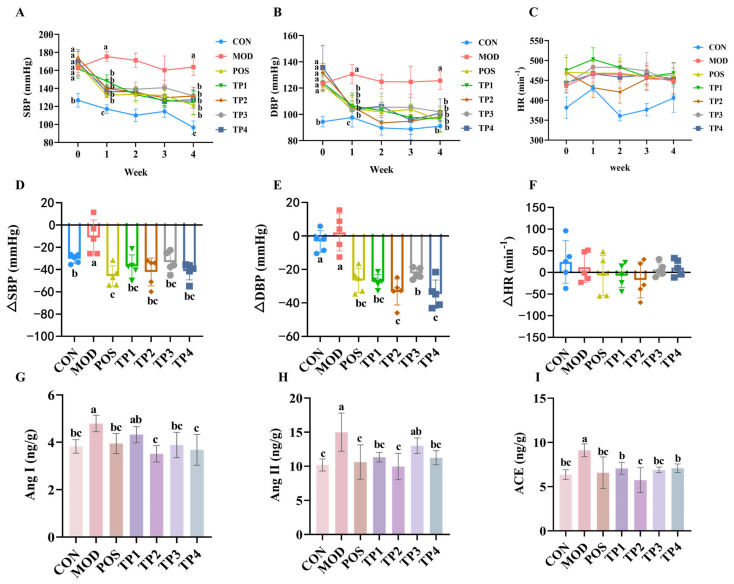
Comparative analysis of green tea peptides on reducing blood pressure, heart rate, and Ang I, Ang II, and ACE levels in kidneys of SHRs (*n* = 5). Levels of (**A**) SBP, (**B**) DBP, and (**C**) HR at weeks 0–4. Comparative analysis of (**D**) SBP difference (ΔSBP), (**E**) DBP difference (ΔDBP), and (**F**) HR difference (ΔHR) in green tea peptide gavaged rats at week 4 versus week 0. Changes in levels of (**G**) Ang I, (**H**) Ang II, and (**I**) ACE in kidneys of rats after four weeks of continuous gavage. Means denoted by different letter indicate significant differences between groups (*p* < 0.05).

Systolic and diastolic blood pressure are important measures of hypertension. As shown in Figure 1A, the SBP of SHRs was high (>150 mmHg) before the beginning of the experiment (week 0), and the SBP values were almost the same among the groups. With daily gavage administration, the SBP of rats in the TPs and positive drug captopril groups started to decrease significantly from the first week, with SBP values in the range of 130–150 mmHg. In the first week of administration, the SBP values of the captopril group and the four green tea peptide groups reached a statistically significant difference (*p* < 0.05) compared to the model group. At week 4 of administration, the greatest decrease in SBP was observed in the captopril and green tea peptide groups (120–130 mmHg). The decrease in SBP reached a highly significant difference in the captopril, TP1, and TP2 groups when compared with the model group (Figure 1D), with SBP differences ranging from 30 to 50 mmHg. In this experiment, the four green tea peptides had a favorable effect on lowering SBP, with TP2 and TP4 being comparable to the positive drug captopril. As shown in Figure 1B, SHRs all had high DBP (120–135 mmHg) before the commencement of the experiment (week 0), and the DBP values were almost the same between the groups. With daily gavage administration, the DBP values of the rats in the groups administered on Thursdays during weeks 1–4 were all significantly lower compared to the model group. By week 4, the DBP in both the captopril group and the group receiving the four green tea peptides was nearly equivalent to that of the normal control WKY. Additionally, the reduction in DBP across all treatment groups was significantly different from that of the model group (Figure 1E), with a decrease ranging from 20 to 40 mmHg. In this experimental range, all four green tea peptides had a good effect on lowering DBP, with TP1, TP2, and TP4 being comparable to the positive drug captopril. There was no notable pattern of change in HR after gavage in all groups of rats from weeks 1–4 of administration, and there was no statistically significant difference observed between the groups (Figure 1C). As indicated by the change in HR from the baseline measurement in the fourth week (Figure 1F), there were no statistically significant differences observed in heart rate variations across the various dosing groups. The findings suggest that the four green tea peptides did not affect the HR of SHRs.

ACE hydrolyzes Ang I to Ang II and increases blood pressure. Following treatment with four different green tea peptides, the levels of Ang I in the kidneys of rats were measured, as shown in Figure 1G. In the SHR group, the levels of angiotensin I were observed to be reduced across all treatment groups in comparison to the model group. The analysis showed that the differences among the captopril, TP1, TP2, and TP3 groups were significant (*p* < 0.05), whereas the differences between the TP1 group and the model group were not statistically significant. Figure 1H shows the levels of Ang II in the rat kidneys, and no statistically significant difference was observed between the TP3 group and the model group. In contrast, significant differences were observed in the TP1, TP2, and TP4 groups (*p* < 0.05). After treatment with the four green tea peptides, the levels of ACE in the kidneys of rats were detected and the results are shown in Figure 1I. Compared with the model group, the levels of ACE in the kidneys of SHRs in all groups were significantly decreased (*p* < 0.05).

The findings indicated that the four peptides derived from green tea markedly decreased blood pressure as well as the concentrations of associated proteins in the kidneys that are responsible for elevating blood pressure in SHRs. The effect of TP2 was the most significant, and the four green tea peptides had no effect on HR in SHRs.

### 3.2. Green Tea Peptide Ameliorated Renal Injury in SHRs

**Figure 2:**
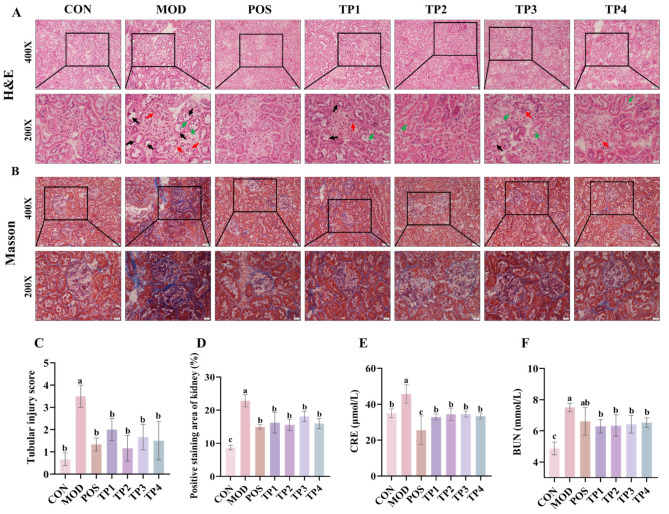
Comparative analysis of green tea peptides in attenuating renal tubular dilatation, glomerular fibrosis injury, and serum CRE and BUN levels in SHRs. (**A**) H&E staining of cortical region of kidney showing tubular damage (black arrow), exposed nuclei (green arrow), and thickened basement membrane (red arrow) (200× and 400×) (*n* = 3). (**B**) Masson’s trichrome staining of renal cortex shows areas of fibrosis (blue) (200× and 400×) (*n* = 3). (**C**) Grading of renal injury (0–4) according to H&E staining results. Scale bar = 20 μm. (**D**) Masson’s trichrome-stained fibrosis area was quantified by ImageJ software. Levels of (**E**) CRE and (**F**) BUN in serum of rats were used to assess degree of renal injury (*n* = 5). Means denoted by different letter indicate significant differences between groups (*p* < 0.05).

Pathological changes in renal function were assessed by analyzing renal tissue using H&E staining and Masson trichrome staining. Pathological changes in H&E staining of SHR kidneys were observed by electron microscopy. In addition, the extent of renal damage was scored according to our previous method (Figure 2C) [34]. In comparison to the normal group, renal tissues in the model group showed significant tubular dilatation (black arrows), cells with naked nuclei (green arrows), and thickening of glomerular basement membranes and thylakoid membranes (red arrows) (Figure 2A). The administration of green tea peptides over four weeks led to a substantial decrease in tubular dilatation and basement membrane thickening in the areas affected by renal lesions in SHRs. Additionally, there was a significant decrease in the tubular injury score (Figure 2C). Furthermore, Masson trichrome staining demonstrated a significant accumulation of collagen fibers in the renal tissues of the model group (Figure 2B) (blue). Calculation of the proportion of the blue region within the selected regions using Image J software (Figure 2D) showed that 4 weeks of treatment with green tea peptides significantly reduced the degree of renal fibrosis caused by hypertension. The above results indicated that all four green tea peptides had significant effects on hypertension-induced renal injury, with TP2 and TP4 being the most effective in alleviating renal injury.

Blood CRE and BUN levels represent the extent of kidney damage. As is consistent with the observed morphological alterations, both blood CRE and BUN concentrations were significantly elevated in SHRs in comparison to the normal group (Figure 2E,F). Furthermore, these levels were significantly decreased to near-normal values following various treatments with green tea peptides (*p* < 0.05). In conclusion, green tea peptides were shown to mitigate renal injury in hypertensive rats and facilitate the restoration of their renal function.

### 3.3. Green Tea Peptides Through Ang II/TGF-β1/SMAD Pathway to Lower Blood Pressure and Attenuated Renal Fibrosis

**Figure 3:**
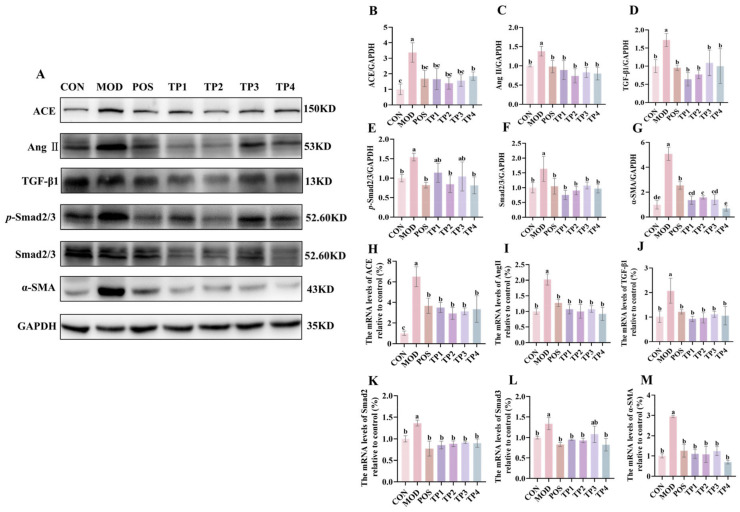
Comparative analysis of green tea peptides on reduction in renal ACE, Ang II, TGF-β1, *p*-Smad2/3, Smad2/3, and a-SMA expression in SHRs (*n* = 3). (**A**) Protein immunoblotting of ACE, Ang II, TGF-β1, *p*-Smad2/3, Smad2/3, and α-SMA in rat kidney tissues. Image J quantified protein expression of (**B**) ACE, (**C**) Ang II, (**D**) TGF-β1, (**E**) *p*-Smad2/3, (**F**) Smad2/3, and (**G**) α-SMA. RT-qPCR analysis of mRNA levels of (**H**) ACE, (**I**) Ang II, (**J**) TGF-β1, (**K**) Smad2, (**L**) Smad3, and (**M**) α-SMA. Means denoted by different letter indicate significant differences between groups (*p* < 0.05).

The renin–angiotensin–aldosterone system (RAAS) plays a crucial role in the maintenance of blood pressure homeostasis in the body. ACE and Ang II serve as key indicators of RAAS activation. We conducted an analysis of the gene and protein expression levels of critical targets within the RAAS. The expression of ACE and Ang II, proteins linked to increased blood pressure, was significantly higher in the kidneys of SHRs compared to WKY (Figure 3B,C). TP treatment resulted in a notable reduction in the protein expression of ACE and Ang II in the kidneys of SHRs compared to the model group. The overall trend for the gene expression of ACE and Ang II was similar to their respective protein expression. The relative expression levels of these genes were markedly elevated in the SHR group; however, this trend was notably attenuated following treatment with captopril and the four groups of green tea peptides, as illustrated in Figure 3H,I. Notably, both at the protein and gene levels, the TP2 group showed a highly significant reduction in the expression of ACE and Ang II in the kidneys.

The activation of RAAS in the kidney further contributes to fibrosis. TGF-β1 mediates fibrotic and inflammatory responses through the Smad signaling pathway, with Smad2/3 being a key molecule involved in TGF-β1 activity, leading to renal fibrosis [35]. We examined the protein and gene expression of TGF-β1, Smad2/3, and *p*-Smad2/3 in renal tissues (Figure 3D–F,J–L). The model group exhibited a marked upregulation in comparison to the normal group, indicating that hypertension may have induced renal abnormalities in the SHR model. However, captopril and green tea peptide treatments reversed this trend and reduced the degree of kidney damage. TGF-β1, which is activated by RAAS, promotes EMT via mechanisms that are both dependent on and independent of SMAD proteins [13]. The expression of α-SMA serves as a phenotypic indicator of myofibroblast activity, which is a feature of late-stage EMT [36]. Consequently, we analyzed the protein and gene expression levels of α-SMA (Figure 3G,M). Compared to the normal group, the model group exhibited a marked increase in the expression of α-SMA at both the protein and gene levels, which was significantly downregulated after treatment with captopril and the four green tea peptides involved in the development of EMT. Among the treatments, TP2 showed the most significant effect on inhibiting renal fibrosis.

Collectively, these findings indicated that green tea peptides might lower blood pressure and mitigate renal fibrosis via the Ang II/TGF-β1/SMAD signaling pathway.

### 3.4. Green Tea Peptide Relieves Kidney Inflammation

**Figure 4:**
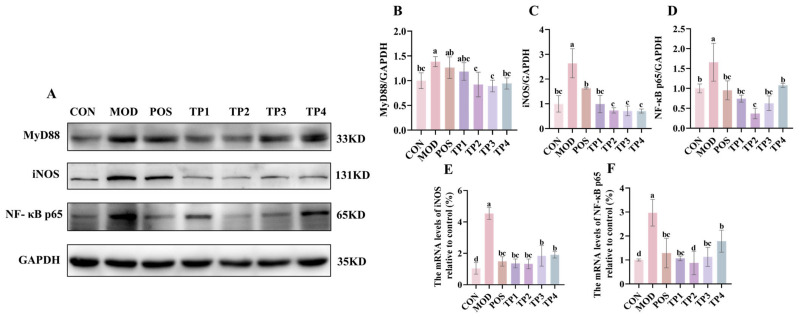
Comparative analysis of green tea peptides on reducing MyD88, iNOS, and NF-kB p65 expression in SHR kidneys (*n* = 3). (**A**) Protein expression levels of MyD88, iNOS, and NF-κB p65 in rat kidneys. ImageJ quantified protein levels of (**B**) MyD88, (**C**) iNOS, and (**D**) NF-κB p65, and mRNA levels of renal (**E**) iNOS and (**F**) NF-κB p65 were analyzed by RT-qPCR. Means denoted by different letter indicate significant differences between groups (*p* < 0.05).

TGF-β1, through the Smad signaling pathway, not only induces fibrosis but also mediates inflammation. The MyD88/NF-κB p65/iNOS signaling pathway plays a key role in the inflammatory response, with activation of NF-κB further promoting an elevation in the concentrations of pertinent inflammatorycytokines [37]. Protein blotting revealed that the expression of MyD88, NF-κB p65, and iNOS was significantly upregulated in the kidneys of SHRs compared to WKY. Treatment with captopril and green tea peptides reversed these changes (Figure 4A–E). Regarding the gene expression of NF-κB p65 and iNOS, the overall trend was similar to the corresponding protein expression. These results suggested that green tea peptides inhibited hypertension-induced activation of the MyD88/NF-κB p65/iNOS signaling pathway in rat kidneys, with TP2 showing the most significant effect on mitigating renal inflammation.

## 4. Discussion

Hypertension-induced renal injury is a pathological condition characterized by renal tubular injury, renal interstitial fibrosis, and glomerulosclerosis, resulting from sustained elevated blood pressure. This leads to alterations in both the structure and function of the kidneys, posing a significant threat to the health of hypertensive patients and serving as a major contributor to ESRD [38]. There are many drugs available for the treatment of renal fibrosis. However, these interventions are frequently associated with negative side effects, including gastrointestinal distress and fatigue [39]. Recently, bioactive peptides derived from food sources, such as milk, egg yolks, and mushrooms, were reported [40,41,42]. Compared to traditional drugs, these food-borne bioactive peptides offer advantages such as high specificity, fewer side effects, wide availability, high potency, and low toxicity [43].

This research investigated the antihypertensive and kidney-protective properties of small molecule peptides derived from green tea in SHRs. Our findings revealed that a four-week continuous treatment regimen resulted in a notable decrease in both SBP and DBP levels. TP2 and TP4 had more pronounced blood pressure-lowering effects than TP1 and TP3. CRE and BUN levels can determine the extent of kidney injury with high sensitivity, specificity, and accuracy [44,45]. In this research, following four weeks of ongoing treatment, all of the above indicators show significant improvement. Masson trichrome staining of the kidneys further revealed the presence of glomerular and interstitial fibrosis. In addition, the green tea peptide treatment was found to reduce this fibrosis. TP2 had the most significant effect in ameliorating kidney injury, and TP1, TP3, and TP4 had similar effects.

Notably, RAAS is strongly associated with fibrosis [46]. ACE, a zinc metallopeptidase that serves a crucial function in regulating peripheral blood pressure through RAAS, hydrolyzes inactive Ang I into the vasoconstrictor Ang II, subsequently resulting in an elevation of blood pressure [47]. Ang II subsequently attaches to its type I receptor (AT1), activating Smads. This process aids in the production and release of the pleiotropic factor TGF-β1, which signals the pro-fibrotic mediator Smad2/3 through a positive feedback loop. Smad2/3 is then recruited to and phosphorylated by the TGF-β1 receptor, ultimately promoting fibrosis [48,49]. In this investigation, we noted a significant elevation in the protein and gene expression levels of TGF-β1, Smad2, and Smad3, as well as an increase in the protein expression of TGF-β1 and phosphorylated Smad2/3 in the renal tissues of SHRs. However, treatment with green tea peptides led to a significant decrease in the expression levels of TGF-β1, Smad2, Smad3, and *p*-Smad2/3. Furthermore, these peptides were found to attenuate EMT by reducing the expression of the pro-fibrotic marker α-SMA at both the protein and gene levels. Increasing evidence from various studies indicates that TGF-β1 plays a crucial role in mediating EMT, and the TGF-β1/Smad3 signaling pathway is essential to the EMT process [50]. Among them, TP1, TP2, and TP4 had comparable inhibitory effects on these indicators, followed by TP3. Inazaki et al. found that inflammation and fibrosis, such as renal interstitial fibrotic areas, collagen deposition, myofibroblast infiltration, and apoptotic cell accumulation, were attenuated by the inactivation of the TGF-β1/Smad3 signaling pathway [51]. Renal injury induced by hypertension is complex, with the core process involving the activation of the renin–angiotensin–aldosterone system (RAAS), along with vascular endothelial dysfunction, oxidative stress, and inflammatory processes [52]. Recent research has indicated that ferroptosis is strongly associated with target organ damage in cardiovascular diseases [53]. The regulatory processes involved in iron-induced cell death are closely linked to those associated with oxidative stress and inflammation. Moreover, iron-associated cell death, along with specific changes such as reduced mitochondrial volume and altered double membrane density, may contribute to kidney injury in cases of salt-sensitive hypertension [54].

In addition, the non-Smad pathway is instrumental in the advancement of renal injury induced by hypertension, mainly through the increased expression of pro-inflammatory factors [55]. The activation of the NF-κB signaling pathway initiates a cascade of inflammatory responses, resulting in the secretion of multiple pro-inflammatory cytokines [56]. The NF-κB protein family comprises multiple members, including RelA (p65), RELB, *C*-REL, among others. It is important to note that each of these proteins contains a transcriptional activation domain (TAD) situated at the *C*-terminus, which facilitates the activation of target gene expression [57]. In this experiment, p65 was chosen as the NF-κB subunit to be studied. The MyD88-dependent signaling pathway serves as a crucial activator of NF-κB p65 and plays a significant role in its subsequent regulatory mechanisms [58]. NF-κB p65 serves as a significant downstream transcription factor that is instrumental in the transcriptional regulation of iNOS. This process subsequently impacts blood pressure and contributes to the aggravation of renal injury [59,60]. In this study, we observed a significant reduction in the protein expression of MyD88, as well as the protein and gene expression levels of NF-κB p65 and iNOS, in the kidneys of SHRs following treatment with green tea peptides. These findings suggest that the protective effect of green tea peptides against renal inflammation in hypertensive rats may be linked to the MyD88/NF-κB p65/iNOS signaling pathway. Among the four green tea peptides, TP2 had the most significant anti-inflammatory effect, while TP1, TP3, and TP4 had comparable effects.

Through in vivo experiments, we determined that the four TPs had significant effects in alleviating hypertension and its renal injury. Still, the effects were variable and might be closely related to their oral bioavailability [61]. Peptide chain length and amino acid composition were reported to significantly affect the digestive stability and absorption efficiency of ACE inhibitory peptides in the gastrointestinal tract [62]. Among the four green tea peptides studied, TP1, TP2, and TP4 with 7-peptide structures exhibited stronger in vivo ACE inhibition than TP3 with 11-peptide structures, which is likely related to the better gastrointestinal tolerance and higher bioavailability of the shorter peptide chains [30,63]. In addition, it was reported that ACE inhibitory peptides are generally short peptides consisting of 2–12 amino acids with a high content of hydrophobic amino acids [64,65]. TP3, on the other hand, has the lowest content of hydrophobic amino acids, which may be the reason for its weaker ACE inhibitory effect. In the primary structure of the peptide, the characteristics of the *C*-terminal amino acids and the *N*-terminal branched aliphatic amino acids have an important influence on ACE inhibitory activity [66]. The *N*-termini of the four TPs in this study contained four aliphatic amino acids, namely, aspartic acid (Asp), valine (Tyr), leucine (Leu), and methionine (Met), which is in agreement with the conclusion of the *N*-terminal conformational relationship reached by previous authors [67]. However, the mechanism of their inhibition of ACE activity is not yet clear. In the future, we can construct a pharmacophore model of green tea ACE inhibitory peptides and further explore the intermolecular interactions and potential binding sites of the four TPs with ACE using molecular docking [68].

In this paper, SHR and WKY were used as experimental models to investigate the in vivo antihypertensive and mitigating mechanisms of hypertensive renal injury effects of four green tea peptides. There are still parts that need to be explored in depth in terms of experimental design. The experiments used a male SHR model, whereas the process of blood pressure regulation in female SHRs may vary depending on the physiological response [69]. In addition, four TPs inhibited hypertension and hypertensive renal injury at the same dose, but designing a dose gradient helped to reveal potential queer effects. Prolonged exposure to hypertension was reported to lead to complications in several organs and systems [70]. In this study, we focused on the protective effect of TPs on kidneys in SHRs, but TPs may also be beneficial in attenuating damage to other organs such as the heart and brain. Currently, the pathogenesis of essential hypertension is unknown, and it is a chronic condition that needs to be controlled by lifelong lifestyle interventions and long-term medication when necessary [71]. Our four-week TP dosing experiment demonstrated good relief of hypertension and its renal injury, but longer dosing enables further assessment of efficacy persistence and potential cumulative toxicity. In future work, we can use both male and female rat models, design dose gradients, focus on different hypertensive injury target organs, and extend the intervention period to comprehensively enhance the clinical translational value of our research.

In clinical practice, combining two antihypertensive drugs with different mechanisms in treating hypertensive disorders can achieve complementary effects and improve therapeutic efficacy. For example, the combination of an angiotensin II receptor blocker (telmisartan) and an angiotensin-converting enzyme inhibitor (ramipril) is beneficial for controlling blood pressure, providing cardiovascular protection, and treating hypertension complications [72]. In addition, combination therapy with angiotensin-converting enzyme inhibitors and diuretics effectively reduces the incidence of cardiovascular complications of hypertension [73]. Therefore, we predict that green tea peptide, as a food-derived ACE-inhibiting peptide with high safety and low toxicity, may be expected to replace drugs with similar mechanisms and be used in combination with other antihypertensive drugs for the clinical treatment of hypertension and hypertensive nephropathy. This idea needs to be verified by further studies.

## 5. Conclusions

In summary, green tea peptides, which are bioactive compounds obtained from natural sources, have the potential to markedly lower blood pressure and mitigate kidney damage associated with hypertension in hypertensive rat models. This effect is achieved through the modulation of the Ang II/TGF-β1/SMAD signaling pathway. Additionally, they also demonstrated anti-inflammatory properties. Therefore, green tea peptides hold great potential as treatment options for kidney injury caused by hypertension.

## Figures and Tables

**Table 1:** Primer sequences of GAPDH, TGF-β1, Smad2, Smad3, ACE, Ang II, α-SMA, NF-κB p65, and iNOS.

Primer Names	Sequences
GAPDH	Forward: 5′-AGACAGCCGCATCTTCTTGT-3′
	Reverse: 5′-CTTGCCGTGGGTAGAGTCAT-3′
TGF-β1	Forward: 5′-ACCTGCAAGACCATCGACATG-3′
	Reverse: 5′-CGAGCCTTAGTTTGGACAGGAT-3′
Smad2	Forward: 5′-TTTGCCGAGTGCCTAAGTGATA-3′
	Reverse: 5′-TTCTTATGGTGCACATTCGAGTC-3′
Smad3	Forward: 5′-GCTGTCTACCAGTTGACTCGCAT-3′
	Reverse: 5′-GGGTGCTGGTCACTGTCTGTCT-3′
ACE	Forward: 5′-TCCTATTCCCGCTCATCT-3′
	Reverse: 5′-CCAGCCCTTCTGTACCATT-3′
Ang II	Forward: 5′-CACCCCTTTCATCTCCTCTACTA-3′
	Reverse: 5′-TCTTGCCTCACTCAGCATCTT-3′
α-SMA	Forward: 5′-AACACGGCATCATCACCAAC-3′
	Reverse: 5′-CACAGCCTGAATAGCCACATAC-3′
NF-κB p65	Forward: 5′-AAGATCAATGGCTACACAGG-3′
	Reverse: 5′-CCTCAATGTCTTCTTTCTGC-3′
iNOS	Forward: 5′-GACCAAACTGTGTGCCTGGA-3′
	Reverse: 5′-TACTCTGAGGGCTGACACAAGG-3′

## Data Availability

The data that support the findings of this study are available from the corresponding author upon reasonable request.

## Abbreviations

The following abbreviations are used in this manuscript:

TP	Tea peptides
SHR	Spontaneously hypertensive rat
WKY	Wistar-Kyoto rat
SBP	Systolic blood pressure
DBP	Diastolic blood pressure
HR	Heart rate
ACE	Angiotensin-converting enzyme
Ang I	Angiotensin I
Ang II	Angiotensin II
CRE	Creatinine
BUN	Blood urea nitrogen
*p*-Smad2/3	Phosphorylated-Smad2/3
Smad2/3	Sma- and Mad-related proteins 2/3
TGF-β1	Transforming growth factor-β1
α-SMA	α-smooth muscle actin
MyD88	Medullary differentiation factor 88
NF-κB p65	Nuclear factor-κB p65
iNOS	inducible nitric oxide synthase
EMT	Epithelial-to-mesenchymal transition
ESRD	End-stage renal disease
RAAS	Renin–angiotensin–aldosterone system
